# Supplementary figures and images for: Dynamic up- and down-regulation of the default (DMN) and extrinsic (EMN) mode networks during alternating task-on and task-off periods
Source: PLoS One. 2019 Sep 19;14(9):e0218358. doi: 10.1371/journal.pone.0218358 (PMC6752853; doi:10.1371/journal.pone.0218358)

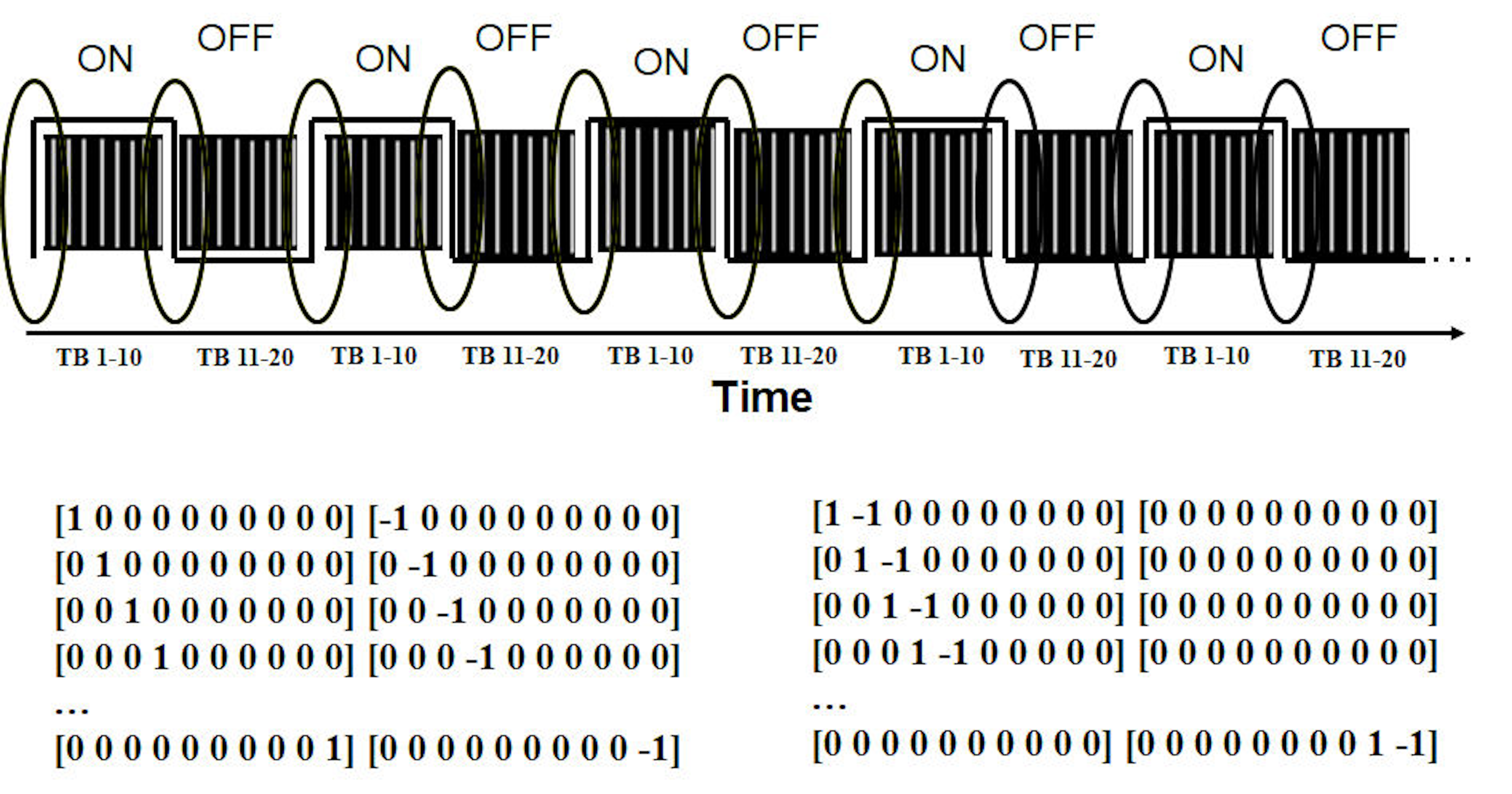

Supplement: S1 Fig — Outline of the block-design with alternating ON-and OFF-periods with corresponding task-processing and resting, respectively. The time-line illustrates the specification of the separate time-bins (TB) for respective ON- and OFF-blocks, with the transition points between blocks marked with circles. The tables under the design illustrates the contrasts used in the analysis for the time-bin (left) and time-derivative (right) analyses, respectively (see text for further details. (TIFF) [file pone.0218358.s001.tiff]
